# Changes of the ocular surface and aquaporins in the lacrimal glands of rabbits during pregnancy

**Published:** 2011-11-09

**Authors:** Chuanqing Ding, Michael Lu, Jianyan Huang

**Affiliations:** Department of Cell and Neurobiology, University of Southern California, Keck School of Medicine, Los Angeles, CA

## Abstract

**Purpose:**

To test the hypotheses that pregnancy represents a physiologic condition that is associated with dry eye symptoms, and the expression of aquaporin 4 (AQP4) and AQP5 are altered in the lacrimal gland (LG) from term pregnant rabbits.

**Methods:**

Schirmer’s test, tear break-up time (BUT), and Rose Bengal staining were used to evaluate ocular surface health. LG were obtained from term pregnant rabbits and age-matched female control rabbits and then processed for laser capture microdissection (LCM), real time RT–PCR, western blot, and immunofluorescence for the detection and quantification of mRNA and proteins of AQP4 and AQP5.

**Results:**

Pregnant rabbits demonstrated typical clinical symptoms of dry eye, including decreased Schirmer score and BUT as well as increased Rose Bengal staining of cornea. In term pregnant rabbits, mRNA for *AQP5* from whole LG was significantly lower than that of control rabbits, while mRNA for *AQP4* was not. Levels of mRNA for *AQP4* and *AQP5* underwent significant changes in acini and epithelial cells from specific duct segments during pregnancy. Western blot from whole LG lysates demonstrated that expression of AQP4 was 24% more abundant in term pregnant rabbits while AQP5 was 22% less when compared to control rabbits respectively. At term pregnancy, AQP4 immunoreactivity (AQP4-IR) was increased in acini while its intensity remained the same in ducts. AQP5-IR was present in both apical and basolateral membranes of acinar cells in normal control and pregnant rabbits, while ductal cells in pregnant rabbits also showed significant amount of AQP5-IR.

**Conclusions:**

The data presented here demonstrated significant dry eye symptoms in pregnant rabbits. Our data also showed altered expressions of *AQP4* and *AQP5* during pregnancy and suggested that these changes may contribute to the altered LG secretion and dry eye symptoms during pregnancy.

## Introduction

Dry eye is one of the most frequently encountered problems for eye clinicians. It affects millions of people with most being women, especially those after menopause [1]. Dry eye involves eye discomfort due to abnormalities in the quantity and/or quality of the tears, with the lacrimal gland (LG) being a major contributor.

There is a general clinical impression that pregnant women more frequently report symptoms of dry eye than non-pregnant women. Our epidemiological study suggests that a subpopulation of pregnant women indeed experience increased symptoms of dry eye during the third trimester of their pregnancies [2]. Because dry eye is closely related to changes in sex hormone milieu in the body [3], substantial changes of the hormone profile during pregnancy may play a role in the occurrence of dry eye.

Our recent report in pregnant rabbits showed that basal LG fluid production from term pregnant rabbits decreased from 0.35±0.04 in control rabbits to 0.21±0.05 μl/min, while pilocarpine stimulated secretion increased from 5.23±0.4 in control rabbits to 11.4±0.56 μl/min [4]. This dramatic increase of pilocarpine stimulated secretion prompted us to hypothesize that this may due to changes of the expression and/or function of aquaporin (AQP) that have recently been reported in the LG and proposed to play a significant role in LG secretion and dysfunction [5-12].

Like other exocrine secretions, LG fluid secretion is an osmotic process driven by the transepithelial secretion of electrolytes and water that is mediated by AQP and ion transporters [5,13-19]. AQP is a group of water channel proteins that are responsible for rapid water transport across plasma membranes in many organisms [20-22]. Thirteen subtypes of AQP have been characterized so far and at least 2 subtypes have been found in LG. Aquaporin 5 (AQP5) is localized to the apical membranes of LG acinar and ductal cells from mice and humans [7-9,23-26], while aquaporin 4 (AQP4) is found on the basolateral membranes of mouse LG acinar and ductal cells [23]. In the rabbit LG, we recently found that AQP4 was expressed in the basolateral sides of acinar and duct cells, while AQP5 was present in both apical and basolateral sides of acinar cells [5,6].

LG fluid is produced in two stages: production of primary fluid in the acini and modification into the final fluid during transit through the duct system before reaching the ocular surface. Most studies regarding the LG have focused on the role of acinar cells, with only a few having paid sufficient attention to the duct system [5,13,14,18,27]. However, increasing evidence supports the notion that the lacrimal duct system plays critical roles in LG secretion.

Although our previous reports demonstrated that pregnant rabbit showed decreased basal LG fluid production [4], there has never been any documentation of ocular surface changes in these animals other than our report in an abstract format [28]. Therefore, the aim of the present study was to test the hypotheses that pregnancy represents a physiologic condition that is associated with dry eye symptoms, and that the abundance of AQP is altered in the LG from pregnant rabbits, with particular emphasis on the duct system.

## Methods

### Animals and tissue preparation

Twelve pregnant and twelve normal control New Zealand White adult female rabbits (Irish Farms, Norco, CA) weighing about 4.0 kg were used throughout our studies. Pregnant rabbits were time dated with day zero corresponding to the date of coitus. Normal gestation in the rabbit is 31 days. Animals were narcotized with a mixture of ketamine (40 mg/ml) and xylazine (10 mg/ml) and given an overdose of Nembutal (80 mg/kg) for euthanasia. Term pregnant rabbits were sacrificed on the 29th day of pregnancy. All studies conformed to the standards and procedures for the proper care and use of animals by the US Public Health Service Policy on Humane Care and Use of Laboratory Animals.

### Ocular surface evaluations

Routine diagnostic techniques used in testing humans for dry eye, i.e., Schirmer’s test, tear break-up time (BUT), and Rose Bengal staining, were used to evaluate the health status of the ocular surface of normal control rabbits, and pregnant rabbits at 2, 3, and 4 weeks of gestation.

1. Schirmer’s test: Schirmer’s strip was inserted in the lower fornix of the eye without anesthesia for 1 min, after which the strip was removed and the length of the wetted area was measured. Every care was taken to avoid touching the cornea.
2. BUT: to evaluate tear film stability, 2 μl of fluorescein was instilled into the lower fornix of the conjunctiva. The rabbit was allowed to blink several times to distribute the fluorescein evenly on the cornea. The time from opening of the eyes to the appearance of the first dry spot in the central cornea was measured 3 times and the mean was recorded.
3. Rose Bengal staining: Rose Bengal stains whenever there is insufficient protection of surface epithelial cells. Rose Bengal solution (2 μl) was placed in the lower conjunctiva. Results were recorded on a cornea diagram and scored using a modified standardized grading system of 0 to 3 [29]. The intensity of staining of the cornea was graded, with the maximum as 3.

### Laser capture microdissection (LCM)

The technique has been described in detail in our previous publications [5,6,30,31]. In brief, after frozen sections were stained with cresyl violet with the LCM Staining Kit (Applied Biosystems, Foster City, CA), areas of interest in tissue sections were laser captured using a PixCell II LCM System (Arcturus Bioscience, Mountain View, CA). About 100 epithelial cells were collected for each acinus and duct segment sample, and six replicate samples of acinar cells and epithelial cells from each duct segment were collected from each animal.

### RNA extraction and reverse transcription

Total cellular RNA was isolated from RNALater-treated samples with RNeasy^®^ midiKit plus on-column DNase digestion to reduce the possibility of DNA contamination (Qiagen, Valencia, CA). RNA quality and quantity was evaluated using a Nanodrop ND-1000 spectrophotometer (Nanodrop Technologies, Wilmington, DE) and 5 μg samples of total RNA samples were then reverse-transcribed to cDNA only if the 260/280 ratio was above 1.9 (High Capacity cDNA Reverse Transcription Kit containing random primers and MultiScribe™ Reverse Transcriptase; Applied Biosystems) according to manufacturer’s instructions.

### Real time RT–PCR analysis and pre-amplification

The sequences of primers and probes of *AQP4* and *AQP5* used in this study are listed in Table 1, which is the same as our previous report [5]. The sequences were selected on computer (Primer Express; ABI) and synthesized by ABI. All probes incorporated the 5′ reporter dye 6-carboxyfluorescein (FAM) and the 3′ quencher dye 6-carboxytertramethylrhodamine (TAMRA).

**Table 1:** Primers and probes used for real-time RT–PCR.

**Gene**	**Sequence**	**Tm (°C)**	**Product Size (bp)**	**Accession #**
***AQP4***				
Forward primer	5′-CGTTTTAAAGAAGCCTTCAGCAA-3′	52.60	72	AF000312
Reverse primer	5′-CCTGTTGTCCTCCACCTCCAT-3′	55.45		
Probe	5′-CTGCCCAGCAAACGAAAGGGAGCTA-3′			
***AQP5***				
Forward primer	5′-GGGCAACCTGGCTGTCAA-3′	54.40	84	AF495879
Reverse primer	5′-AGCTGGAAGGTGAGGATCAACTC-3′	55.95		
Probe	5′-CTCAACAACAACACGACACCGGGC-3′			

For LCM samples, pre-amplification was performed using TaqMan^®^ PreAmp Master Mix Kit (Applied Biosystems). The pooled assay mix was prepared by combining up to 50 of 20× TaqMan^®^ Gene Expression Assays into a single tube and using nuclease-free water to dilute the pooled assays to a final concentration of 0.2×. The 50 μl pre-amplification reaction included 25 μl of 2× TaqMan^®^ PreAmp Master Mix, 12.5 μl of 0.2× pooled assay mix, and 12.5 μl of cDNA sample. The reactions were incubated in the DNA Engine® Thermal Cycler for 10 min at 95 °C followed by 14 cycles at 95 °C for 15 s and 4 min at 60 °C and then held at 4 °C. The pre-amplification product was then diluted 1:20 with 1× TE buffer and analyzed by TaqMan^®^ real time RT–PCR.

Amplification was performed with an ABI PRISM® 7900HT Sequence Detection System (Applied Biosystems) using TaqMan^®^ Gene Expression Master Mix (Applied Biosystems) containing the internal dye ROX as a passive reference. The PCR reaction volume was 10 μl and contained 1× TaqMan^®^ Gene Expression Master Mix, 300 nM forward and reverse primers, 250 nM probes, and 50 ng of cDNA template. The FAM signal was measured against the 6-carboxy-X-rhodamine (ROX) signal to normalize for non-PCR-related fluorescence fluctuations. The cycle threshold (C_T_) value represented the refraction cycle number at which a positive amplification reaction was measured and was set at 10× the standard deviation (SD) of the mean baseline emission calculated for PCR cycles 3–15. Each sample was measured in triplicate. The difference between the C_T_ values for each target mRNA and for the internal housekeeping gene glyceraldehyde 3-phosphate dehydrogenase (*GAPDH*) in each sample was used to calculate the abundance of target mRNA relative to the abundance of *GAPDH* mRNA in the same sample.

### Immunofluorescence (IF) and confocal microscopy

Primary antibodies used were purchased from Santa Cruz Biotechnology (Santa Cruz, CA). The dilution for AQP4 (goat polyclonal, C-19) was 1:50, and 1:40 for AQP5 (goat polyclonal, C-19). The secondary antibodies used were fluorescein isothiocyanate (FITC)-conjugated AffiniPure donkey anti-goat IgG (Jackson ImmunoResearch Laboratories, West Grove, PA) at a dilution of 1:200.

Samples frozen in Optimal Cutting Temperature (OCT) compound were cut 8 μm thick and placed on slides then fixed with ready-to-use formaldehyde/zinc fixative (Electron Microscopy Sciences, Hatfield, PA) for 15 min. They were then washed in phosphate-buffered solution (PBS) 3× for 10 min each and blocked with donkey normal serum (Jackson ImmunoResearch Laboratories) for 1 h at room temperature. The slides were then incubated with primary antibodies at respective dilutions overnight at 4 °C. On the next day, slides were again washed 3× for 10 min in PBS and incubated with secondary antibody for 1 h at room temperature then washed 3× for 10 min in PBS and 1× for 15 min in 4 mM sodium bicarbonate. Finally, one drop of aqueous mounting medium (Vector Laboratories, Burlingame, CA) was placed on slides and covered with coverslip. Slides were observed with a Zeiss LSM 710 confocal laser scanning microscope (Carl Zeiss Microimaging, Thornwood, NY). FITC-conjugated secondary antibodies were visualized by excitation at 488 nm using an argon laser. Images were analyzed with LSM image browser and PhotoShop (Adobe Systems, Mountain View, CA).

### Western blot

LG samples were homogenized in isolation buffer (5% sorbitol, 0.5mM disodium EDTA, 0.2 mM phenylmethylsulfonyl fluoride, protease inhibitor cocktail, 5 mM histidine-imidazole buffer, pH 7.5), and centrifuged at 2,000× g for 20 min. The supernatants were denatured in SDS–PAGE sample buffer for 20 min at 60 °C, resolved on a 4%–20% gradient SDS–PAGE gel (Bio-Rad, Hercules, CA), and then transferred onto PVDF (Immobilon-P; Millipore, Billerica, MA). To assess AQP proteins, a constant amount of protein from each sample was analyzed. Membrane blots were probed with AQP4 at the dilution of 1:250, and AQP5 at 1:1,000. All blots were incubated with Alexa 680-labeled donkey anti-goat secondary antibody (Molecular Probes, Eugene, OR) and detected with an Odyssey Infrared Imaging System (Li-Cor, Lincoln, NE). Densitometry analysis of resulting gel was performed by the manufacturer’s software.

### Statistics

For mRNA data from whole gland samples, unpaired *t*-tests were performed. Relative mRNA abundance data from LCM samples and densitometry results of western blots were subjected to ANOVA (ANOVA) with SigmaPlot 11.0 (Systat Software, San Jose, CA).

## Results

### Ocular surface evaluations

**Figure 1:**
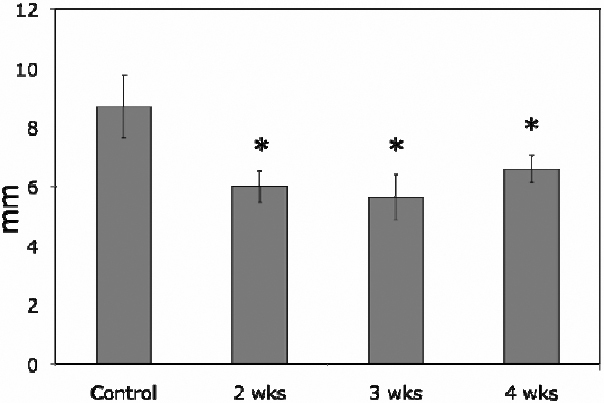
Schirmer tests of normal control and pregnant rabbits. Schirmer score was 8.69±1.07 mm in normal control rabbits while the scores were reduced to 6±0.54 mm at 2 weeks’ pregnancy, 5.63±0.77 mm at 3 weeks, and 6.59±0.45 mm at 4 weeks pregnancy, with the score at each time point of pregnancy was significantly lower than that of normal controls (p<0.05, as indicated by *). Data are presented as mean±standard error of the mean (SEM).

In normal control rabbits, the Schirmer test score was 8.69±1.07 mm, while they were significantly decreased at each time point of gestation, i.e., 6±0.54 mm at 2 weeks, 5.63±0.77 mm at 3 weeks, and 6.59±0.45 mm at 4 weeks (p<0.05, Figure 1).

**Figure 2:**
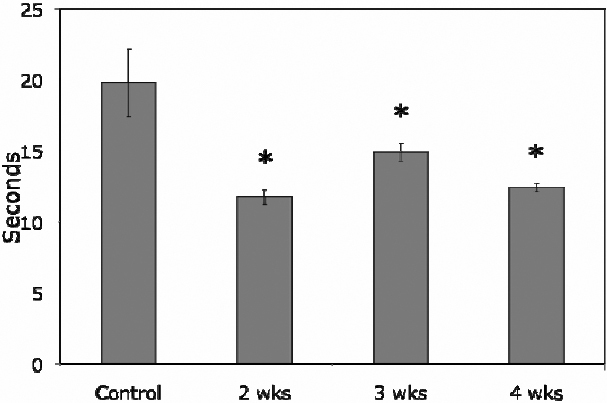
BUT was 19.79±2.39 s in normal control rabbits and was reduced to 11.75±0.51 s at 2 weeks’ pregnancy, 14.9±0.65 s at 3 weeks, and 12.43±0.31 s at 4 weeks pregnancy. Each time point of pregnancy was significantly lower than that of normal control (p<0.05, as indicated by *). Data are presented as mean±SEM.

BUT was 19.79±2.39 s in normal control rabbits and the scores were significantly reduced in pregnant rabbits to 11.75±0.51 s at 2 weeks, 14.9±0.65 at 3 weeks, and 12.43±0.31 s at 4 weeks’ pregnancy (p<0.05, Figure 2).

**Figure 3:**
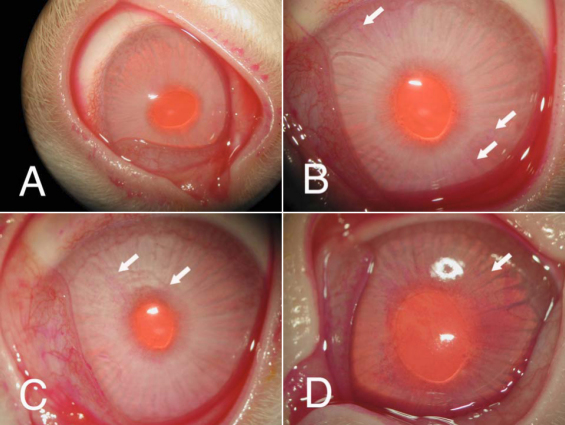
Representative images of Rose Bengal staining of rabbit corneas. The test was unremarkable in normal control rabbits (**A**), but starting at 2 weeks of pregnancy, punctate staining (arrows) was observed in the corneas of many rabbits (**B**). At 3 weeks of pregnancy, the rabbit cornea typically shows a moderate staining (**C**, arrows). Moderate to severe staining was found in approximately 50% of pregnant rabbits at 4 weeks pregnancy (**D**). As shown in panel **D**, one rabbit’s cornea showed prominent punctate staining in every quadrant of the cornea.

**Figure 4:**
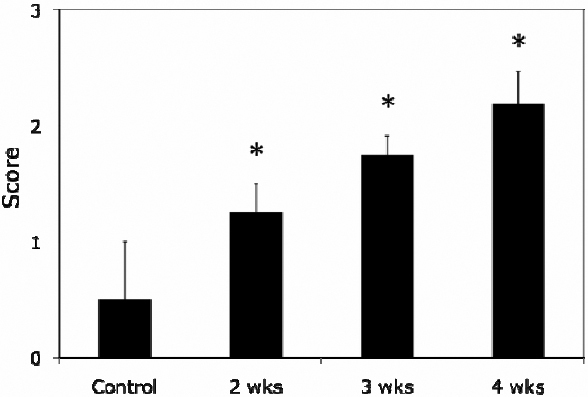
Grading of Rose Bengal staining. The scores increased from 0.5±0.5 in controls to 1.25±0.25 of 2 weeks’ pregnancy, and 1.75±0.16 of 3 weeks, and 2.19±0.28 of 4 weeks. Data are presented as mean±SEM.

Rose Bengal test was unremarkable in most corneas of normal control rabbits, but in pregnant rabbits, punctate staining started to show up in many rabbits, and the intensity and severity increased as the pregnancy progressed. At term, there was a moderate to severe staining in approximately 50% of pregnant rabbits, with punctate staining observed in every quadrant of the cornea (Figure 3D). Grading of the scorings demonstrated a progressive increase of the staining (Figure 4).

Although Schirmer and BUT results for pregnant rabbits were both decreased starting at 2 weeks and continued at 3 and 4 weeks, the Rose Bengal test showed a gradual increase of staining as the pregnancy progressed from 2, 3, and 4 weeks of pregnancy. Therefore, we decided to use rabbits at 4 weeks of pregnancy, i.e., term pregnancy, for the following studies.

### Expression of *AQP* mRNA

**Figure 5:**
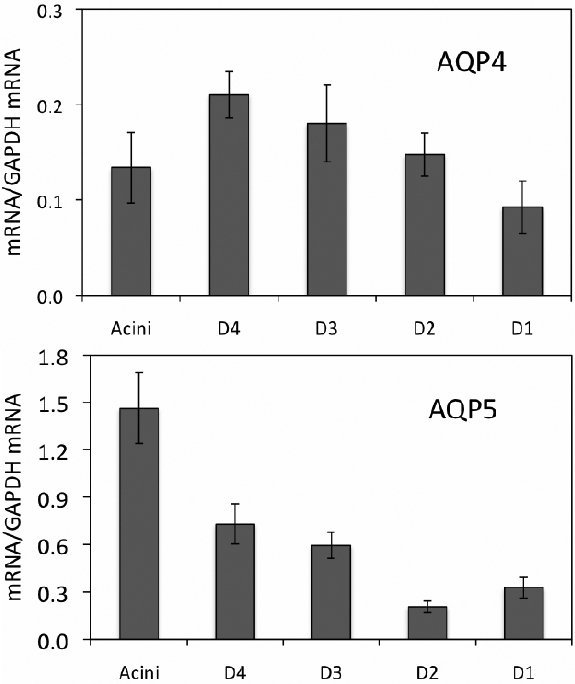
Real-time RT–PCR of *AQP4* and *AQP5* from LCM samples of term pregnant rabbits. Table 2 compares these results to that of normal rabbit controls. mRNA levels for *AQP4* was the lowest in interlobar duct, rather than acini as observed in control rabbits [5], and its level was significantly lower in all duct segments except interlobular duct (p<0.05). **AQP5** mRNA was the highest in acini and was significantly lower (p<0.05) than that of normal control rabbits [5]. However, mRNA abundance was significantly increased in intralobular, interlobular, and interlobar ducts (p<0.05), while no change was found in intralobar duct. D4: intralobular duct. D3: interlobular duct. D2: intralobar duct. D1: interlobar duct. Data are presented as mean±SEM of 3 animals.

mRNA level for *AQP4* from whole LG of term pregnant rabbits was 0.07266±0.00714, which is not significantly different from that of normal control rabbits (0.06753±0.00798), as we reported recently [6]. However, mRNA levels from epithelial cells collected by LCM (Figure 5) showed significant decrease in all duct segments except interlobular duct and acini (Table 2), as compared to those from normal control rabbits [5].

**Table 2:** mRNA changes from term pregnant rabbits as compared to control rabbits [5].

**Gene**	**Whole LG**	**Acini**	**Intralobular**	**Interlobular**	**Intralobar**	**Interlobar**
*AQP4*	−	−	↓	−	↓	↓
*AQP5*	↓	↓	↑	↑	−	↑

Note: ↓: significant decrease; ↑: significant increase; −: no significant difference.

Contrary to *AQP4*, mRNA level for *AQP5* from whole LG of term pregnant rabbits (5.51±0.33) was significantly lower than that from normal controls (7.1±0.24), as we reported [6], representing a 22.3% decrease. In epithelial cells from pregnant rabbits LG collected by LCM, *AQP5* mRNA was significantly lower in acini while its level was significantly increased in every duct segment except intralobar duct (Figure 5, Table 2), as compared to those from normal control rabbits [5].

### Western blot

**Figure 6:**
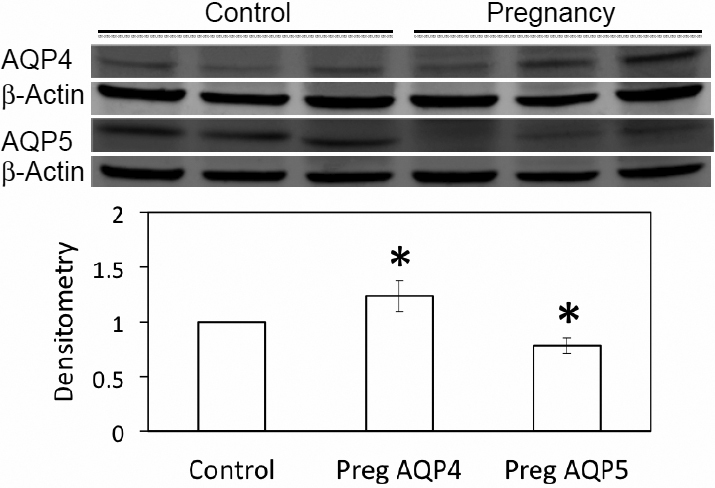
Western blots of AQP4 and AQP5 from whole LG homogenates. AQP4 was significantly increased in LG from pregnant rabbits, while AQP5 was decreased (p<0.05). β-Actin was used as loading control. Data are representative images of at least 3 different animals each.

Western blots of whole LG lysates demonstrated the expressions of AQP4 and AQP5 (Figure 6). Densitometry analysis indicated that AQP4 from term pregnant rabbits was 24% more than normal control rabbits, while AQP5 expression was 22% less (p<0.05).

### Immunofluorescence

**Figure 7:**
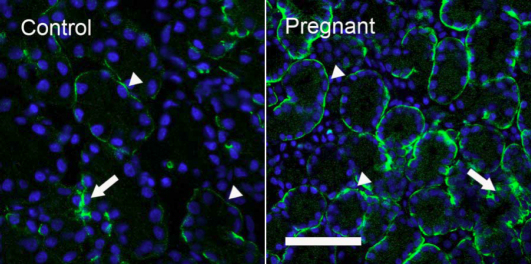
Immunofluorescence of AQP4-IR. Control: AQP4-IR was observed on the basolateral sides of acinar and duct cells, with duct (arrow) showing much stronger AQP4-IR than acini (arrowheads), results in accordance with our previous reports [5,6]. Pregnant: Acini in pregnant rabbits showed substantially stronger basolateral staining (arrowheads) than control, whereas AQP4-IR in ducts (arrow) appeared to be similar to that of control. In both images, DAPI was used to stain nuclei as bright blue to demonstrate the morphologic profiles of acini and ducts. Scale bar=50 μm.

AQP4 immunoreactivity (AQP4-IR) was observed on the basolateral membranes of acini and ducts in LG of both control and pregnant rabbits, with ducts showing stronger AQP4-IR than acini (Figure 7). AQP4-IR in ducts from pregnant animals did not differ significantly from ducts from controls, whereas its intensity was much stronger in the acini of pregnant rabbits than acini from control animals, and more acini in pregnant rabbits showed stronger AQP4-IR than control rabbits.

**Figure 8:**
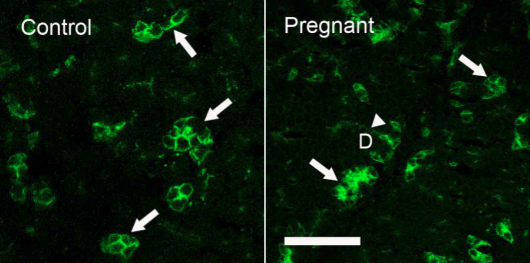
Immunofluorescence of AQP5-IR. Control: AQP5-IR was present in both basolateral and apical membranes of acinar cells, and distributed among acini in a “mosaic” pattern, with some acini and/or acinar cells demonstrating much stronger AQP5-IR (arrows) than the rest of acini/acinar cells. However, little AQP5-IR was detected in duct cells. These results were similar to our previous reports [5,6]. Pregnant: as in control LG, AQP5-IR was also present in a “mosaic” pattern with a similar intensity and distribution pattern (arrows). However, in contrast to control animals, ductal cells also exhibited a significant amount of AQP5-IR (arrowhead). D=duct. Scale bar=50 μm.

AQP5-IR was found in apical and basolateral membranes of acinar cells and was distributed among acini in a “mosaic” pattern with some acini and/or acinar cells in both control and pregnant rabbits exhibiting much stronger AQP5-IR than other acini/acinar cells (Figure 8). Minimal AQP5-IR was detected in ductal cell membranes of control animals, while significant AQP5-IR was observed in ducts of pregnant rabbits, results similar to those we recently reported in rabbits with induced autoimmune dacryoadenitis [6].

## Discussion

Dry eye has been linked to many risk factors, including altered sex hormones, especially androgens [3,32-34]. During pregnancy, there are significant fluctuations of hormonal profiles that may influence the functional status of LG and therefore pose as a risk factor for dry eye. Indeed, previous studies have shown that the rabbit LG undergoes an immunophysiological transformation during pregnancy, similar to that of the mammary gland as it prepares to deliver milk–the ductal epithelial cell prolactin immunoreactivity increases and redistributes from the apical to the basolateral cytoplasm [35,36]. During pregnancy, infiltrated lymphocytes in the rabbit LG disperse to the interacinar space from their normal periductal foci [4,35].

We have found that many pregnant rabbits demonstrated typical clinical symptoms of dry eye, including decreased Schirmer’s test scores and BUT, and increased Rose Bengal staining. These results corroborate our epidemiology study in pregnant women [2] and our previous report that demonstrated pregnant rabbits’ basal LG fluid production was decreased while pilocarpine stimulated secretion rate increased and protein concentration decreased [4]. Recent reports indicate that women with one or more pregnancies also have an increased risk of having Sjögren's syndrome [37,38], an autoimmune disease that frequently involves pathologies in salivary glands and LG that result in dry mouth and dry eye.

AQP are membrane proteins responsible for rapid water transport across plasma membranes, following osmotic gradients generated by ion transport proteins. The detection of AQP4 and AQP5 in the rabbit LG, which confirmed our previous reports in rabbits [5,6], mouse [23,39], rat [40,41], and humans [7], suggests that they may play a role in LG secretion.

At term pregnancy, our results demonstrated that the expression patterns of AQP4 and AQP5 in the LG undergo significant changes at both gene and protein levels in both acini and specific duct segments. The largest change happened in acini and interlobar duct, where the mRNA levels of *AQP4* in interlobar duct and *AQP5* in acini from pregnant rabbits were only 1/3 that of normal rabbits. Significant changes of AQP in LG have been suggested to play a role in dry eye [7,8]. Tsubota et al. [7] reported that in the LG of Sjögren’s syndrome patients, AQP5 was found to congregate within the cytoplasm rather than being transported to the membranes, which suggests that misprocessing of AQP5 might be involved in the pathogenesis of Sjögren’s syndrome, despite contrasting report from other researchers [10]. AQP have also been found in tears in mouse with dacryoadenitis, suggesting its leakage from LG epithelial cells [24]. In mouse LG, it was reported that in response to pilocarpine-induced LG secretion, AQP5 was increased at the apical membrane of acinar cells [23].

The significant increase of AQP5-IR in ductal cells of pregnant rabbits suggests increased potential for water transport across both apical and basolateral membranes. Although water flow could be bidirectional, i.e., either increased secretion or increased reabsorption from the ductal cells, the reduced basal LG final fluid secretion from pregnant animals [4] is indicative of increased reabsorption of the primary fluid by ductal cells, while increased final LG fluid secretion in response to pilocarpine stimulation during pregnancy suggests increased secretion from ductal cells. However, because mRNA for AQP4 was decreased in duct cells and mRNA for AQP5 was decreased in acinar cells during pregnancy, numerous combinations of altered water transport in acinar and duct cells could occur, as reported before [7,10,23,24].

In accordance with our previous reports in normal control rabbits [5] and rabbits with induced autoimmune dacryoadenitis [6], AQP4 are preferentially located in ducts while AQP5 in acini from LG of pregnant rabbits, despite their significant changes during pregnancy. It is worth noting that the relative abundance of mRNA of *AQP5* was about 76 fold that of *AQP4* in the whole LG, suggesting that AQP5 is the dominant subtype of AQP in the LG. These data are in support of the critical role of AQP in lacrimal secretion, particularly in the ducts, which are in accordance with previous findings regarding lacrimal ducts’ active role in LG fluid production [5,14,18,27]. While AQP4 and AQP5 are two distinctive isoforms of AQP, both of them are primarily permeable to water [20,21]. However, their preferential distribution in acini and ducts suggests that they play different roles in acinar and ductal cells, and their changes during pregnancy also suggest their potential site-specific involvement in pregnancy-related LG deficiency.

It should be noted that there are substantial discrepancies in mRNA and protein expressions of the data presented here. Several mechanisms, i.e., redistribution changes of proteins between cell membranes and intracellular stores during pregnancy [19,42-45], changes of AQP recycling [46], and changes in protein redistribution and recycling during inflammation [19,46], could explain these apparent discrepancies. Similar discrepancies have been reported in previous studies that demonstrated that many mRNA expression differences are not reflected at the protein levels [47,48]. Furthermore, it should be noted that differences in protein expressions may not always correspond with differences in their functional status. This topic is beyond the scope of the present study but highlights the necessity of functional studies to elucidate the functional changes of LG during pregnancy.

Although it has been demonstrated extensively that AQP play critical role in water transport in many epithelia, care should be taken in considering how big the role it may be playing in the LG. Unlike salivary glands, which have secretion rates as high as ~75–1,000 ml per day in humans [49] and AQP are heavily involved, the average tear production is only ~5 ml per day in human [49] and water secretion can be sufficiently achieved by AQP-independent water transport by glandular epithelial cells, which means AQP are not required at physiologic conditions in some epithelia when the secretion rate is low, i.e., LG [25,26,50] and sweat gland [51]. Therefore, it has been suggested that when secretory rate is low, AQP are not required at physiologic conditions [25,50]. However, AQP may play a critical role when the secretion rate is high and their changes may contribute to the altered LG secretion in Sjögren’s syndrome [6], pregnancy [4], and the ocular surface changes during pregnancy as we documented here.

In summary, our studies have shown increased dry eye symptoms in pregnant rabbits. The fact that AQP4 and AQP5 expressions underwent significant changes during pregnancy suggests changes in their functional status which may potentially contribute to the changes of LG fluid secretion and dry eye symptoms during pregnancy. These data also support our previous findings that both acini and ducts contribute to LG secretion. Further functional studies are needed to identify the changes in each duct segment as well as in the acini during pregnancy.
